# Cortical Microhemorrhages Cause Local Inflammation but Do Not Trigger Widespread Dendrite Degeneration

**DOI:** 10.1371/journal.pone.0026612

**Published:** 2011-10-19

**Authors:** Nathanael L. Rosidi, Joan Zhou, Sanket Pattanaik, Peng Wang, Weiyang Jin, Morgan Brophy, William L. Olbricht, Nozomi Nishimura, Chris B. Schaffer

**Affiliations:** 1 Department of Biomedical Engineering, Cornell University, Ithaca, New York, United States of America; 2 Department of Chemical and Biomolecular Engineering, Cornell University, Ithaca, New York, United States of America; Virginia Commonwealth University, United States of America

## Abstract

Microhemorrhages are common in the aging brain, and their incidence is correlated with increased risk of neurodegenerative disease. Past work has shown that occlusion of individual cortical microvessels as well as large-scale hemorrhages can lead to degeneration of neurons and increased inflammation. Using two-photon excited fluorescence microscopy in anesthetized mice, we characterized the acute and chronic dynamics of vessel bleeding, tissue compression, blood flow change, neural degeneration, and inflammation following a microhemorrhage caused by rupturing a single penetrating arteriole with tightly-focused femtosecond laser pulses. We quantified the extravasation of red blood cells (RBCs) and blood plasma into the brain and determined that the bleeding was limited by clotting. The vascular bleeding formed a RBC-filled core that compressed the surrounding parenchymal tissue, but this compression was not sufficient to crush nearby brain capillaries, although blood flow speeds in these vessels was reduced by 20%. Imaging of cortical dendrites revealed no degeneration of the large-scale structure of the dendritic arbor up to 14 days after the microhemorrhage. Dendrites close to the RBC core were displaced by extravasating RBCs but began to relax back one day after the lesion. Finally, we observed a rapid inflammatory response characterized by morphology changes in microglia/macrophages up to 200 µm from the microhemorrhage as well as extension of cellular processes into the RBC core. This inflammation persisted over seven days. Taken together, our data suggest that a cortical microhemorrhage does not directly cause significant neural pathology but does trigger a sustained, local inflammatory response.

## Introduction

Recent clinical evidence suggests that cortical microhemorrhages, while not producing acute stroke symptoms, are linked to increased risk of cognitive decline and dementia [1], [2]. Traditional animal models of hemorrhage, such as infusion of whole blood or injection of bacterial collagenase, have provided a means to study the physiological impact of large-scale intracerebral hemorrhages (ICH), which affect macroscopic regions of the cortex and do cause acute stroke [3]. However, these methods are unable to reproduce the microhemorrhages that likely stem from the rupture of a single small arteriole or capillary and may underlie aspects of cognitive decline. Due in part to this lack of good animal models, there remains an incomplete understanding of the acute and chronic cellular dynamics and pathophysiological events following microhemorrhage, making progress on therapeutic strategies difficult.

There are multiple potential mechanisms, identified in previous studies of large hemorrhages that may play a role in injuring brain cells after microhemorrhage. Acutely, increases in intracranial pressure and compression of brain tissue resulting from hematoma formation can mechanically injure cells [4] and can crush surrounding blood vessels, reducing cerebral blood flow (CBF) and causing ischemic pathology [5]. The high concentration of glutamate in the blood plasma that is pushed into the brain can cause excitotoxic injury to neurons [6]. Over several days after ICH, additional injury to brain cells can be caused by oxidative stress and free radical damage from, for example, the byproducts of red blood cell (RBC) lysis, as well as by edema formation [7]. Hemorrhage also drives an inflammatory cascade, leading to microglia response and invasion of neutrophils and macrophages, which may exacerbate injury [8].

In this paper, we studied the time course and spatial extent of the pathological consequences of a cortical microhemorrhage in mice. We ruptured individual, targeted arterioles in the cortex using femtosecond laser ablation, producing microhemorrhages [9] that are ∼10,000 times smaller than those produced with existing ICH models [10] and that match the size of the microhemorrhages histologically observed in human brains [11]. We used two-photon excited fluorescence (2PEF) microscopy to track the acute and chronic effects of these microhemorrhages with micrometer-scale spatial resolution. We first quantified the extravasation dynamics of RBCs and blood plasma after microhemorrhage and determined that clotting limits the extent of bleeding. We then measured tissue compression caused by the hematoma and showed this compression was not sufficient to collapse nearby capillaries, although blood flow speeds in these vessels were slightly reduced. Next, we examined structural changes in dendrite morphology, acutely and up to two weeks after microhemorrhage, and found no evidence of large-scale degenerative changes in the structure of the dendritic arbor. The lack of dendrite degeneration we observed is consistent with previous results where extravasation of blood plasma into brain tissue at the border of an ischemic lesion did not lead to dendrite damage over several hours [12]. Finally, we found microglia/macrophages responded within an hour after the microhemorrhage and increased in density near the lesion over one week. This inflammatory reaction did not change in animals that lacked the fractalkine chemokine receptor, CX_3_CR1, suggesting that microglia/macrophages are activated through other signaling pathways after a microhemorrhage. These data suggest that the pathological impact of a microhemorrhage from a single cortical blood vessel may be more dependent on inflammation-mediated injury rather than on direct neuronal degeneration.

## Methods

### Ethics statement

All animal procedures were approved by the Cornell University Institutional Animal Care and Use Committee (protocol numbers 2006-0044 and 2009-0043) and were conducted in strict accordance with the recommendations in the Guide for the Care and Use of Laboratory Animals published by the National Institutes of Health.

### Transgenic animals

We used 25 adult C57BL/6 mice (∼5 to 8 months of age, both sexes, 21–32 g in mass) expressing yellow fluorescent protein (YFP) in a subset of cortical neurons (YFP-H line; stock# 3782; The Jackson Laboratory) [13] for experiments to characterize bleed dynamics, tissue compression, and dendrite health after microhemorrhage. Six adult C57BL/6 mice expressing green fluorescent protein (GFP) in a subset of cortical neurons (GFP-M line; stock #7788; The Jackson Laboratory) were used for experiments tracking the health of individual dendrites after a microhemorrhage. Data on microglia/macrophage recruitment after microhemorrhage was generated using C57BL/6 mice heterozygous (CX_3_CR1^+/GFP^ (4 mice)) and homozygous (CX_3_CR1^GFP/GFP^ (5 mice)) for a knock-in that replaces the CX_3_CR1 fractalkine receptor with GFP, leading to fluorescent labeling of resident microglia cells in the brain as well as in peripheral blood monocytes (CX_3_CR1-GFP; stock# 5582; The Jackson Laboratory) [14]. Three additional heterozygous CX_3_CR1-GFP mice were used to assess the microglial response from a parenchymal laser lesion. Wild-type animals used for quantification of capillary blood flow changes after microhemorrhage (7 mice) in histology (6 mice) and heparin-infusion experiments (3 mice) were age-matched, non-transgenic littermates of the YFP-H animals.

### Chronic cranial imaging window preparation

To prepare a chronic cranial window for imaging [15], mice were anesthetized using 5% isoflurane (VetOne) and maintained at 1.5–2%. Body temperature was kept constant at 37.5°C with a heating blanket and thermometer (50–7053P; Harvard Apparatus). Glycopyrrolate (0.5 mg/kg mouse) (Baxter Healthcare Corp.) was administered intramuscularly while ketoprofen (5 mg/kg mouse) (Fort Dodge) and dexamethasone sodium phosphate (0.2 mg/kg mouse) (American Regent, Inc.) were both administered subcutaneously prior to surgery. A 5-mm diameter circular bilateral craniotomy was performed over the parietal cortex. An 8-mm No. 1.5 glass cover slip (50201; World Precision Instruments) was then placed over the exposed brain and glued to the skull using cyanoacrylate (Loctite 495; Henkel) and dental cement (Co-Oral-Ite Dental Mfg Co.). Animals were administered 5% (wt/vol) glucose in physiological saline (1 ml/kg mouse) subcutaneously and gradually transitioned off isoflurane anesthesia. Mice were then administered ketoprofen (5 mg/kg mouse) every 24 hr for 72 hr and allowed a minimum of five days recovery before *in vivo* imaging and laser-induced microhemorrhaging.

### Two-photon excited fluorescence microscopy

Before imaging sessions, mice were anesthetized with isoflurane (1.5–2%) and retro-orbitally injected with 0.1-mL of 2.5% (wt/vol) neutral (D1830; Invitrogen) or lysine-fixable (D1864; Invitrogen) Texas-Red dextran (70 kDa) in physiological saline to fluorescently label the blood plasma. Animals were transferred to a custom-built two-channel 2PEF microscope with 645/65 emission filters for Texas-Red and 517/65 filters for GFP and YFP. We used a 1045-nm, 1-MHz, 350-fs pulse train from a Yb-fiber oscillator/amplifier system (µJewel FCPA, IMRA America, Inc.) as the excitation source to image dendrites and blood vessels in YPF-H mice, while a 920-nm, 87-MHz, 100-fs pulse train from a Ti:sapphire laser oscillator (MIRA HP; Coherent), pumped by a continuous wave laser (Verdi-V18; Coherent), was used to image GFP and Texas-Red. All 2PEF *in vivo* images were analyzed using ImageJ (NIH), and all images were processed using a one pixel radius median filter.

### Penetrating arteriole microhemorrhage by femtosecond laser ablation

We identified penetrating arterioles (PAs), i.e. arterioles that branch from the surface arteriole network and dive into the brain to feed capillary beds, as target vessels [16]. Before inducing a microhemorrhage, 2PEF image stacks spaced 1-µm apart in the axial direction were taken. The diameter and blood flow speed [17] of the target PA were also measured. Microhemorrhages were produced in the descending segment of the target PA ∼20–100 µm below the surface of the brain in most experiments, or at a depth of ∼300–500 µm for studies of the impact of a deep microhemorrhage on dendrite degeneration. To induce a microhemorrhage, femtosecond laser pulses were tightly focused (20X, 0.95 numerical aperture, water immersion objective; XLUMPlanFl (Olympus)) on the outer edge of the lumen of the target PA [9]. We used 50-fs duration laser pulses produced by a Ti:sapphire regenerative amplifier (Legend 1 k USP; Coherent), pumped by a Q-switched laser (Evolution 15; Coherent) and seeded by a Ti:sapphire oscillator (Chinhook; Kapteyn-Murnane Laboratories Inc.) that was pumped by a continuous wave laser (Verdi-V6; Coherent, Inc). Briefly, a 10-pulse burst (1-kHz repetition rate) with an energy of about 500 nJ/pulse was applied. If the vessel did not rupture, the laser energy was increased by about 50%, and the vessel was irradiated again. This process was repeated until extravasation of RBCs and blood plasma into the parenchyma of the brain was observed. The required laser energy to trigger a microhemorrhage depended principally on the depth of the target vessel beneath the brain surface and the presence of large blood vessels on the surface of the brain above the target vessel. For each microhemorrhage, we used the minimum laser energy required to rupture the target vessel wall and initiate bleeding into the brain. 2PEF images of the bleeding dynamics were acquired over ∼1 min after the microhemorrhage. In addition, 2PEF image stacks of dendrites or microglia/macrophages along with the vasculature were taken 0.5 and 1.5 hours, then one, two, and seven (and 14, in some experiments) days after each microhemorrhage.

### Characterization of RBC and plasma bleeding dynamics

2PEF movies of the bleeding dynamics after microhemorrhage were captured at 3.34 frames/s at the axial location where the vessel was ruptured for ∼1 min with lateral optical resolution of ∼1 µm (n = 14 microhemorrhages). During bleeding, we measured the diameter of the growing disk of fluorescently-labeled blood plasma, as well as the diameter of the growing core of non-fluorescent RBCs, which appeared as dark patches in the sea of fluorescence (>4 measurements at different angles at each time point). At 0.5 and 1.5 hours after microhemorrhage, the RBC core and plasma extravasation diameters were measured from a 20-µm maximum intensity projection of a 2PEF image stack, centered at the axial depth of the microhemorrhage. In some cases, we used tiled image stacks that were later stitched together to increase the area around the hemorrhage we could analyze.

In addition, in three wild-type C57BL/6 mice we intravenously infused heparin before and during a microhemorrhage (n = 10 microhemorrhages). Prior to microhemorrhage induction, a jugular vein catheter was implanted and a 50-µL bolus of heparin (100 U/kg mouse, diluted in 500 µL saline) was infused. This was followed by a constant infusion of the heparin solution at 15-µL/min using a syringe pump (PHD2000; Harvard Apparatus). During the period of steady infusion, target PAs were located and ruptured, and the bleeding dynamics quantified.

### Quantification of tissue displacement after microhemorrhage

To calculate the tissue displacement after microhemorrhage, 2PEF images of fluorescently-labeled dendrites were analyzed using custom-written Matlab (The Mathworks, Inc) software that determined how far dendrites in the post-hemorrhage image shifted laterally compared to the pre-hemorrhage image. The 2PEF images were normalized by first subtracting background (average of the lowest 1% of pixel values) and then dividing by the maximum signal (average of the highest 1% of pixel values). We calculated the dendrite displacement by finding the shift along the *x-* and *y-*axes of a 50×50 pixel region of the post-hemorrhage image that maximized the normalized cross-correlation with the pre-hemorrhage image. To speed up computation and avoid artifacts due to correlation with unrelated dendrites, we considered only a 100×100 pixel region of the pre-hemorrhage image, centered on the post-hemorrhage region. The analyzed regions of the pre and post images were then shifted in ten pixel increments and the algorithm repeated until the dendrite displacement from all regions in the image was determined. Tissue displacement was radially averaged and displayed as a function of distance from the center of the microhemorrhage. For dendrites within about 50 µm from the edge of the microhemorrhage, the shape and orientation of the dendrites changed too much for a cross-correlation based algorithm to reliably determine tissue displacement. In this region, we manually identified the same dendrites before and after the microhemorrhage and measured their displacement. We verified overlap of manual and automated measurements between 100 and 150 µm from the edge of the microhemorrhage.

### Modeling of tissue compression by a microhemorrhage

To describe the compression of brain tissue around a microhemorrhage, we adapted a model from Basser [18] that describes tissue displacement during fluid infusion into the brain. Briefly, we treated the tissue as a fluid-saturated poroelastic network in which the pores are too small to let RBCs into the tissue. The stress-strain relation for the fluid phase is written as
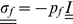
(1)


where 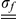 is the stress in the fluid, 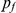 is the pressure in the fluid phase, and 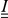 is the unit tensor. For the solid phase, the stress-strain relation is written as
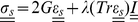
(2)


where 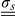 is the stress in the solid, *G* and 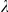 are Lame constants for the solid phase, and *Tr* indicates the trace operation. The strain tensor, 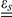, is the symmetric part of the gradient of the displacement vector, 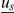, of the solid phase,
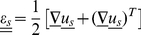
(3)


where *T* is the transpose operation. At mechanical equilibrium, the divergence of the total stress is zero,
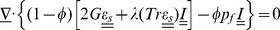
(4)


where 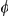 is the tissue porosity. Substituting Eq. (1) into Eq. (4), we obtain Navier's equation,
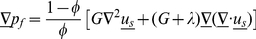
(5)


At mechanical equilibrium, 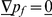. Assuming spherical symmetry, the *r*-component of this equation can then be simplified to
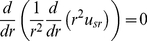
(6)


where *u_sr_* is the displacement of the solid phase in the radial direction. Integrating Eq. (6) twice, we find
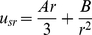
(7)


where *A* and *B* are unknown integration constants. As *r* approaches infinity, *u_sr_* should be finite, implying *A* = 0. At *r* = *a_RBC_*, where *a_RBC_* is the steady-state radius of the cavity filled with RBCs after the microhemorrhage, the stress in the solid phase should equal the contact stress, 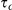, at the boundary between the surrounding tissue and the RBC core,
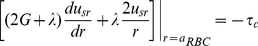
(8)


This boundary condition leads to
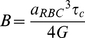
(9)


The radial tissue displacement, *u_sr_*, as a function of the center of the microhemorrhage, *r,* can then be written as:
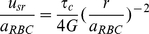
(10)


The unknown contact stress, 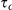, was then determined by fitting Equation (10) to the experimentally measured tissue displacement.

### Quantification of changes in capillary perfusion and blood flow speed

Using 2PEF image stacks of fluorescently-labeled blood plasma, we identified individual capillary segments within 125 µm from the target PA at baseline, then relocated these same segments 1.5 hr after inducing a microhemorrhage. In addition, we classed these segments as flowing or stalled based on the motion of unlabeled RBCs observed over ∼3 s of image frames from the 2PEF stack. In separate animals, we quantified the RBC flow speed in individual capillary segments before and after the microhemorrhage by acquiring line-scan data [17]. In control experiments, we waited the same interval that would be required to produce a microhemorrhage, then remeasured the capillary flow speeds.

### Analysis of dendrite degeneration after microhemorrhage

Using 2PEF image stacks in YFP-H mice taken acutely and out to one week after microhemorrhage, we classified individual dendrites within 150 µm of the target PA as intact or degenerated based on the presence of dendrite blebs (i.e. circular swellings along the dendrite path) [19]. We counted all dendrites in sub-regions of 2PEF image stacks taken from the same volumes within 150 µm of the center of the microhemorrhage across all time points in each animal (n = 24 microhemorrhages in 16 mice). Approximately 10–40 dendrites were identified in each sub-region and each dendrite was classed as showing evidence of degeneration or not. Control regions were at least 1.5-mm away from the microhemorrhage or on the contralateral side. The dendrite exclusion diameter was determined by measuring the distance between the closest dendrites along a line through the center of the microhemorrage core (average of 4 measurements). In the more sparsely-labeled GFP-M mice, we tracked individual dendrites over multiple time points out to two weeks after the microhemorrhage and classified the dendrites as intact, degenerated, or missing as compared to baseline. At each imaging session, the same dendrites were identified.

### Photothrombotic stroke model

A 532-nm continuous wave laser (Compass 215 M, Coherent, Inc.) was added to the 2PEF microscope so that the green light was focused through the microscope objective at the imaging plane [17]. After chronic window implantation and recovery, animals were anesthetized, a photosensitizer dye, rose bengal (RB) (0.03-mg/g mouse diluted at 10-mg/ml in saline), was retro-orbitally injected, and clotting was initiated by focusing ∼5-mW of 532-nm laser light onto the target PA at the surface of the brain for five minutes. Dendrite degeneration was analyzed as described above.

### Analysis of microglia/macrophage dynamics after microhemorrhage

We manually counted the number of microglia/macrophage cell bodies as a function of distance from the center of the microhemorrhage using 2PEF image stacks taken acutely and out to one week after the lesion for heterozygous and homozygous CX_3_CR1-GFP mice. We also identified microglia/macrophages that responded to the microhemorrhage by analyzing the polarity of cellular processes. A cell was determined to be responding if more than 50% of the processes were directed, within a 30-degree cone, toward the center of the microhemorrhage. The microglia/macrophage exclusion diameter was measured as the distance between the closest cellular processes along a line through the center of the microhemorrhage core (average of four measurements per hemorrhage). The maximum distance of microglia/macrophage response was defined as the distance from the center of the microhemorrhage to the farthest cell that directed the majority of its processes towards the lesion site (average of 4–8 measurements per hemorrhage).

### Post-mortem histology

We performed histology seven days after rupturing individual PAs in six C57BL/6 mice (n = 16 microhemorrhages located within the first 100 µm of the brain surface). Animals were transcardially perfused with 15 ml of phosphate buffered saline (PBS) (Sigma-Aldrich) and 30 ml of 4% (wt/vol) paraformaldehyde (Fisher Scientific) in PBS. The brain was extracted from the skull and cryoprotected by immersion in 30% (wt/vol) sucrose in PBS for 24 hr and then in 60% (wt/vol) sucrose in PBS. To facilitate identification of individual microhemorrhages in histological sections, fiducial marks were made at known locations relative to the microhemorrhage sites by insertion of a 30-gauge needle into the brain. The tissue was frozen and cut into 25-µm thick coronal sections on a cryostat and mounted onto microscope slides (Superfrost Plus, Fisher Sci.).

To label RBC breakdown products and neurons, we used Prussian blue and cresyl violet staining, respectively. Brain sections were dehydrated in a graded series of ethanol, immersed in deionized water (dH_2_O), then incubated in a 1∶1 solution of 20% hydrochloric acid and 10% potassium ferrocyanide. Sections were rinsed with dH_2_O and incubated with cresyl violet solution (5% in dH_2_0), then rinsed with dH_2_O, dehydrated in a graded series of ethanol, immersed in xylene, mounted using Permount (Fisher Scientific) and a coverslip was attached. Sections were imaged on a white-light microscope (Axio Examiner; Zeiss).

To visualize astrocyte activation, we immunostained for glial fibrillary acidic protein (GFAP) [20]. Brain sections were rinsed in 0.5% TritonX-100 (Sigma-Aldrich) before overnight incubation with a primary mouse monoclonal anti-GFAP antibody (G3893; Sigma) in a 1:400 dilution in PBS. Sections were then rinsed with PBS and incubated with a 1∶500 dilution of a secondary fluorescein isothiocyanate (FITC)-conjugated donkey anti-mouse IgG antibody (Jackson ImmunoResearch) in PBS. Fluorescent images were collected on a wide-field fluorescent microscope (Axio Examiner; Zeiss) from sections with both primary and secondary antibody, as well as sections containing only the secondary antibody, using identical exposure settings. No staining was observed in sections where the primary antibody was not applied.

### Statistical analysis

A p-value of less than 0.05 was considered statistically significant. Data were tested for normality using a Lilliefors two-sided goodness-of-fit test. For comparisons of effect size between groups, the Mann-Whitney U test was used on data populations that were non-parametric, while a Student's t-test was performed on parametric data. To compare differences in variance between groups, the Ansari-Bradley test was used. The Pearson Product-Moment correlation coefficient was used for statistical correlations on all non-parametric data while an analysis of covariance was used on all parametric data populations.

## Results

We used 2PEF imaging to visualize bleeding dynamics, tissue displacement, and blood flow changes immediately following the microhemorrhage of single PAs in the cortex of mice, and followed the inflammatory response and changes in dendrite structure out to seven to fourteen days after the lesion. The target PAs had a diameter of 19+/−7 µm (mean +/− standard deviation) and a centerline RBC speed of 11+/−6 mm/s. Microhemorrhages were produced by focusing femtosecond-duration laser pulses, 100 to 1500 nJ in energy, on the edge of the vessel lumen of the descending segment of a PA [9]. Nonlinear absorption of laser energy ruptured the vessel wall, which enabled RBCs and blood plasma to enter the brain parenchyma.

### Bleeding of a ruptured cortical penetrating arteriole produced a ∼100-µm diameter hematoma, while blood plasma penetrated five times farther into the brain

**Figure 1:**
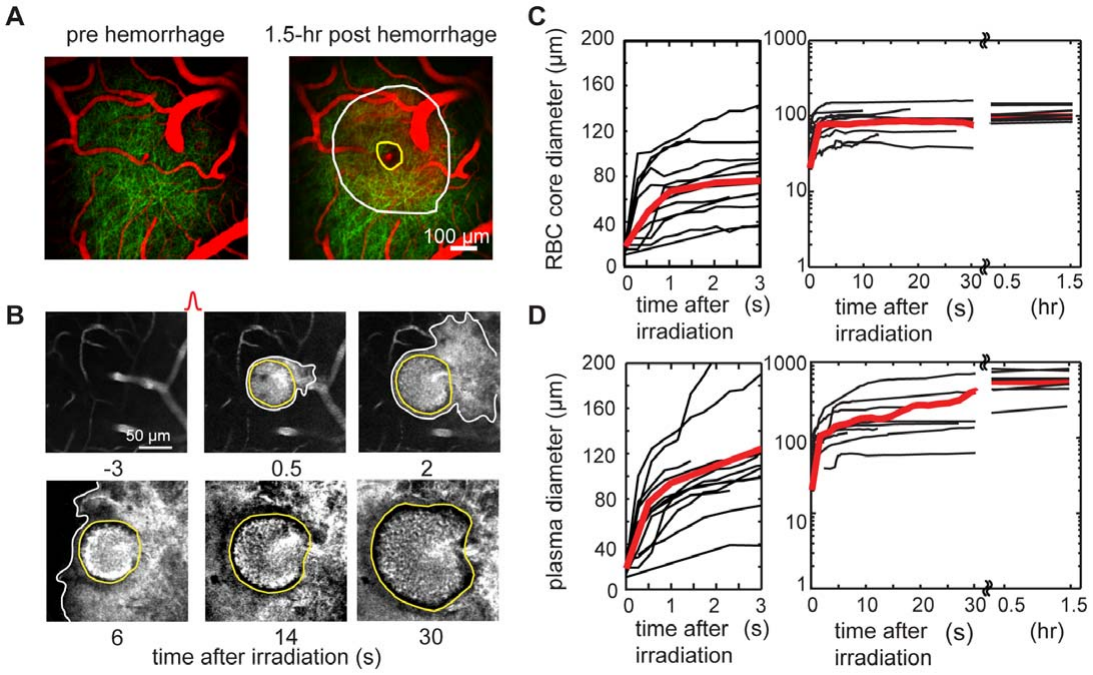
Dynamics of RBC and blood plasma extravasation after laser-induced microhemorrhage. (**A**) Maximum intensity projection of a 2PEF image stack of cortical dendrites (green) and blood vessels (red), before and 1.5 hr after microhemorrhage. The spatial extent of the RBC core (blood plasma) is represented by a yellow (white) outline. (**B**) 2PEF imaging of bleeding dynamics after rupture of a single PA. A RBC-filled core (yellow outline) and diffuse plasma (white outline) expanded into the parenchyma after PA irradiation. (**C**) RBC core and (**D**) blood plasma diameter as a function of time after microhemorrhage.

Immediately after the rupture of a single PA, RBCs and blood plasma exited the lumen of the vessel and entered the brain parenchyma (Figure 1B). The region immediately surrounding the target PA filled with densely packed RBCs that largely excluded the fluorescently-labeled blood plasma and thus appeared dark in the 2PEF image. This RBC core was surrounded by a ring of fluorescence, indicating extravasated blood plasma (Figure 1A) [9]. The diameter of the RBC core expanded to 78+/−23 (mean+/− standard deviation; 14 hemorrhages in 11 mice) within 3 s and stabilized at 100+/−31 µm after 0.5 hrs (Figure 1C). The blood plasma entered further into the parenchyma, reaching a diameter of 119+/−50 within 3 s, continued rapid expansion over 30 s, and stabilized at a diameter of 504+/−237 µm after 0.5 hr (Figure 1D; 13 hemorrhages in 11 mice). The bleeding time, characterized as the time required to reach half the final diameter, was 0.66 s for RBCs and 17 s for blood plasma. In all microhemorrhages, the targeted vessel remained flowing, indicating that a clot obstructing the vessel lumen did not form. In repeated imaging experiments over one week (see below), we never observed any evidence of repeat bleeding of the ruptured vessel.

### Hematoma size was limited by clotting

**Figure 2:**
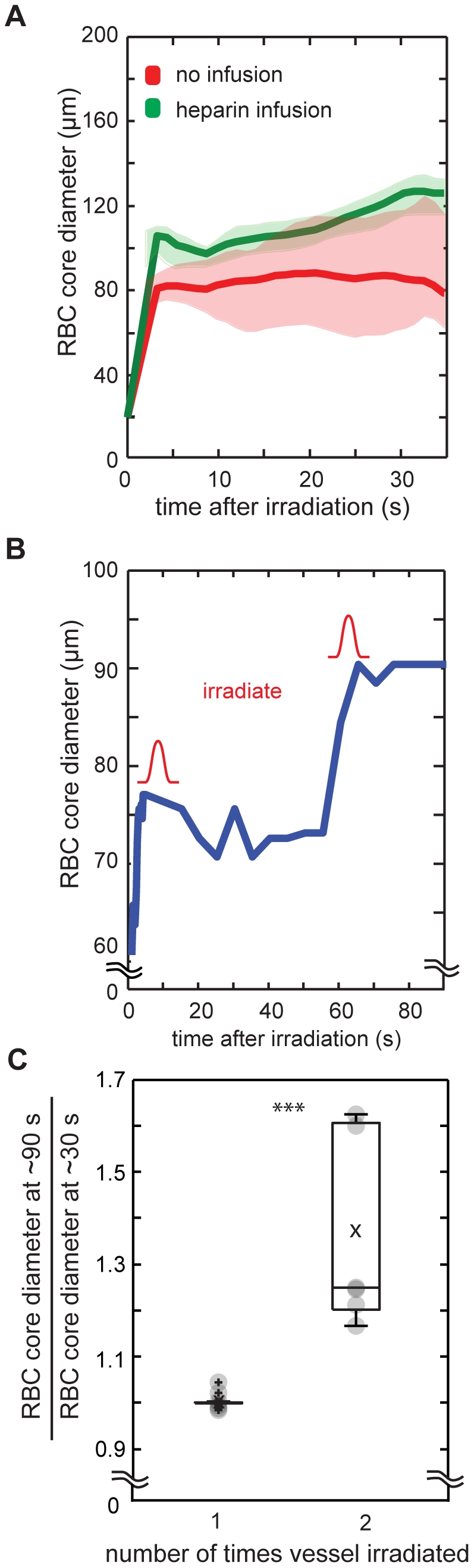
Microhemorrhage size limited by clotting of vessel wall. (**A**) Median RBC core diameter as a function of time for mice receiving heparin infusion (green) and controls (red). Bold lines are running medians with 95% confidence intervals indicated by shaded areas. (**B**) RBC core diameter as a function of time for a PA that was irradiated twice (indicated by red pulses). (**C**) RBC core diameter measured at ∼90 s divided by RBC core diameter measured at ∼30 s for vessels irradiated either at 0 s (one irradiation) or at 0 s and ∼60 s (two irradiations). ***p<0.001; Mann Whitney U test.

To understand what limited the size of the microhemorrhage, we intravenously administered the anticoagulant heparin and ruptured PAs. We compared the RBC core diameter measured 30 s after microhemorrhage in animals with and without heparin administration. We found that heparin increased the hematoma size by 56% to 125+/− µm compared to controls (80+/−20 µm) (Figure 2A; p<0.006; student t-test). In addition, heparin doubled the RBC bleeding time to 1.37 s (p<0.016; Mann-Whitney U test) (10 microhemorrhages in 3 mice). In separate experiments that did not use heparin, we irradiated the target PA a second time after the RBCs stopped expanding (Figure 2B) and observed that the RBC core diameter grew, on average, by 37% after the second irradiation (Figure 2C; p<0.001; Mann-Whitney U test) (6 microhemorrhages in 4 mice). Over the same interval, there was, on average, no growth in the RBC core size for vessels irradiated only once (Figure 2C) (13 microhemorrhages in 11 mice). The larger average RBC diameter with heparin and the increase in RBC core size after a second irradiation suggested that clotting of the vessel wall was a dominant limiting factor in microhemorrhage size. The ∼25 times longer bleeding time for blood plasma as compared to RBCs suggests that the clot may be initially porous, enabling blood plasma (but not RBCs) to exit the vessel.

### Microhemorrhages compressed surrounding tissue

**Figure 3:**
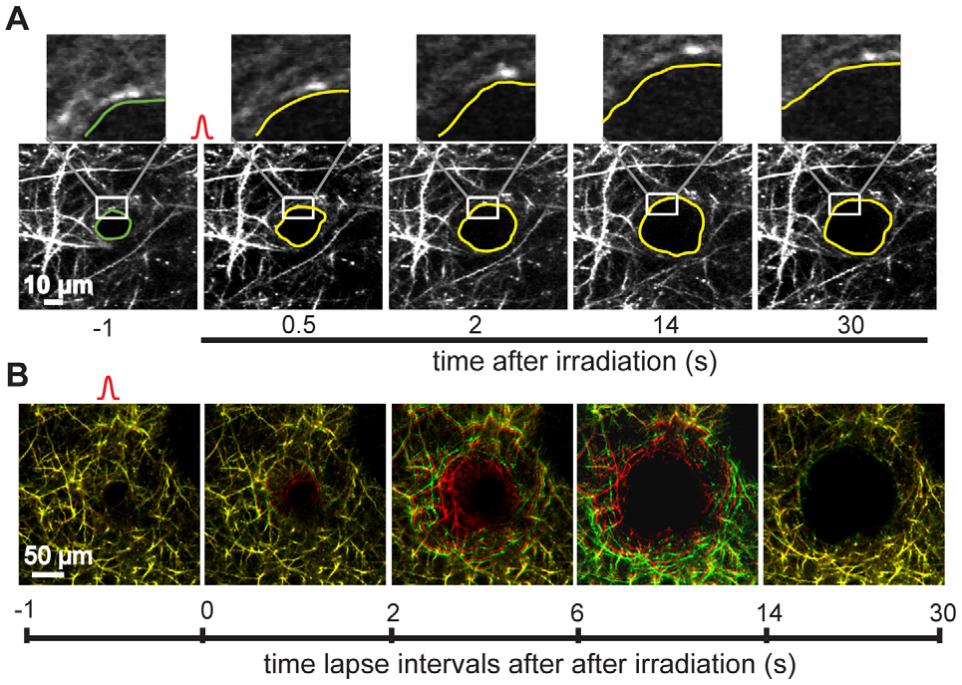
Dendrite displacement by microhemorrhage. (**A**) 2PEF imaging of dendrites after rupture of single PA reveals that dendrites were displaced by extravasating RBCs. Insets track displacement of an individual dendrite that initially abuted the target PA. The green circle in the frame at -1 s represents the size of the target PA, while the yellow outlines in the subsequent frames denote the edge of the RBC core, as determined from simultaneously-acquired 2PEF images of fluorescently-labeled blood plasma. (**B**) Merged time-lapse images of dendrites surrounding the target PA. Red (green) represents the early (late) time point indicated at the bottom left (right) of each frame. Yellow dendrites indicate no displacement over the time interval.

The RBCs that extravasated into the brain after a microhemorrhage are too large to move through the parenchyma. Instead the RBCs compressed the tissue around the ruptured vessel, displacing nearby dendrites (Figure 3). For most dendrites we could trace their displacement until the RBC core stabilized (e.g. all dendrites in Figure 3A and insets). For a small minority of dendrites that were located immediately adjacent to the target PA, the fluorescence rapidly decreased and we were not able to continue to follow them (e.g. red dendrites with no corresponding green dendrites in the second panel of Figure 3B). This decrease in fluorescence may be due to absorption of the emitted light by the RBCs or may represent damage to a small number of dendrites next to the target PA by either the microhemorrhage or the femtosecond laser irradiation used to rupture the vessel.

**Figure 4:**
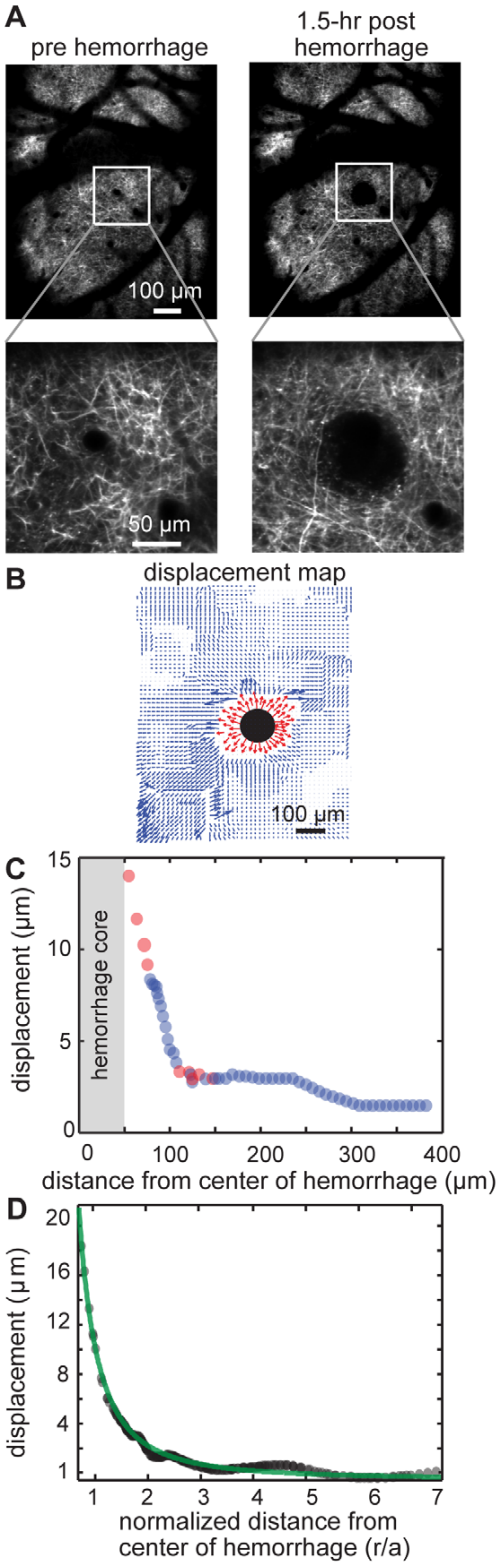
Compression of brain tissue near a microhemorrhage. (**A**) Maximum intensity projections of 2PEF image stacks of cortical dendrites before and 1.5 hr after a microhemorrhage. (**B**) Tissue displacement map representing the magnitude and direction of dendrite displacement after the microhemorrhage shown in panel A. Manual (red) and automated (blue) measurements are both shown. (**C**) Radially-averaged dendrite displacement as a function of distance from the center of the microhemorrhage for the example in panels A and B. (**D**) Average dendrite displacement (black circles) and fit to Equation (10) (green line) as a function of normalized distance from the center of the microhemorrhage.

We analyzed the spatial extent and magnitude of the compression of tissue surrounding a microhemorrhage by the extravasation of RBCs. We used 2PEF images of the apical dendrites of layer V pyramidal neurons (Figure 4A) to measure the direction and magnitude of the tissue displacement due to RBC and plasma extravasation as a function of distance from the microhemorrhage (Figure 4B). A maximum tissue compression of around 20 µm was observed at the edge of the RBC core, which decreased to a few micrometers within 50 µm from the edge of the RBC core (Figure 4C). To group tissue compression data across multiple lesions (10 microhemorrhages in 8 mice), we defined a normalized distance from the center of the microhemorrhage by dividing the distance from the center of the microhemorrhage, *r,* by the RBC core radius, *a_RBC_*, for each microhemorrhage. Fitting the average tissue displacement as a function of this normalized distance to Equation (10) (Figure 4D), we found the contact stress between the RBC core and the surrounding tissue to be 1.5 kPa, assuming a shear modulus of brain tissue, *G*, of 2 kPa, and taking the average RBC core radius to be 50 µm (Figure 4D) [18].

### Microhemorrhages did not collapse nearby capillaries, but did cause modest blood flow speed reductions

**Figure 5:**
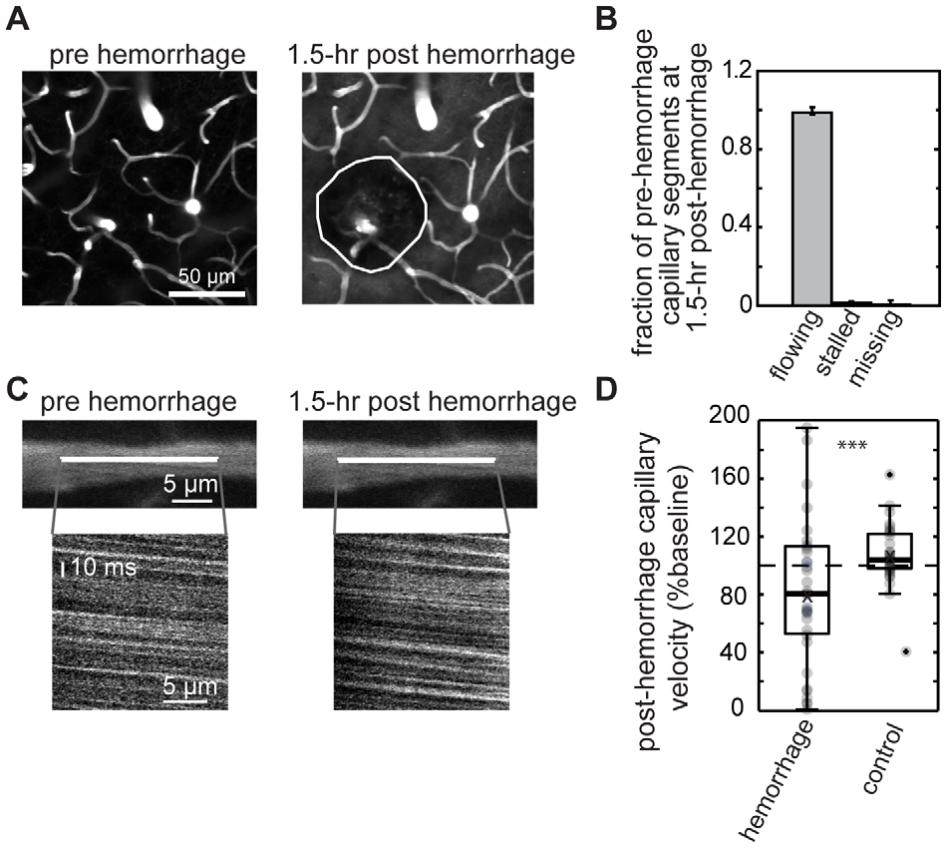
Microhemorrhages do not crush nearby capillaries, but blood flow speed is reduced. (**A**) Maximum intensity projections of 2PEF image stacks of blood vessels in the vicinity of the RBC core (yellow outline) before and 1.5 hr after a microhemorrhage. (**B**) Classification of capillary segments within 125 µm from the target PA, identified before the lesion, as flowing, stalled, or missing at 1.5 hr after the lesion. Error bars represent binomial 95% confidence intervals. (**C**) 2PEF images and space-time linescans of a capillary located ∼70 µm from the target PA before and 1.5 hr after a microhemorrhage. (**D**) Boxplot of capillary blood flow speed 1.5 hr after a microhemorrhage, expressed as a fraction of the baseline speed for capillaries within 125 µm from the target PA. In control measurements, no hemorrhage was produced. ***p<0.001; Mann Whitney U test.

To determine if the tissue compression around the hematoma caused nearby capillaries to collapse, potentially causing ischemia, we identified individual capillary segments within 125 µm of the target PA in image stacks taken before and 1.5 hr after the microhemorrhage (Figure 5A). We classed these segments as stalled or flowing based on the motion of RBCs within the lumen. Of 240 capillary segments (11 micorhemorrhages in 8 mice) only one capillary was missing and three capillaries were stalled after the microhemorrhage (Figure 5B), indicating that microhemorrhages did not lead to a substantial reduction in the number of flowing capillaries in the nearby brain tissue.

In separate experiments, we measured by blood flow speed in individual capillary segments within 125 µm of the target PA at baseline and 1.5 hr after the microhemorrhage (Figure 5C). We found that the median RBC flow speed decreased by 20% after a hemorrhage, while no change was observed in control experiments (Figure 5D; p<0.001, Mann Whitney U test) (35 (36) capillaries across 4 (3) mice with microhemorrhages (controls)). In addition, we find that the variance of the capillary blood flow speeds is about five times larger after a microhemorrhage as compared to controls (p<0.0001, Ansari-Bradley test). Despite the dramatic increase in the variability of capillary flow speed, the decrease in average blood flow speed is modest and so is not likely to lead to ischemic injury to nearby brain cells.

### No acute or chronic dendrite degeneration was observed after microhemorrhage, and displaced dendrites relaxed back over several days

**Figure 6:**
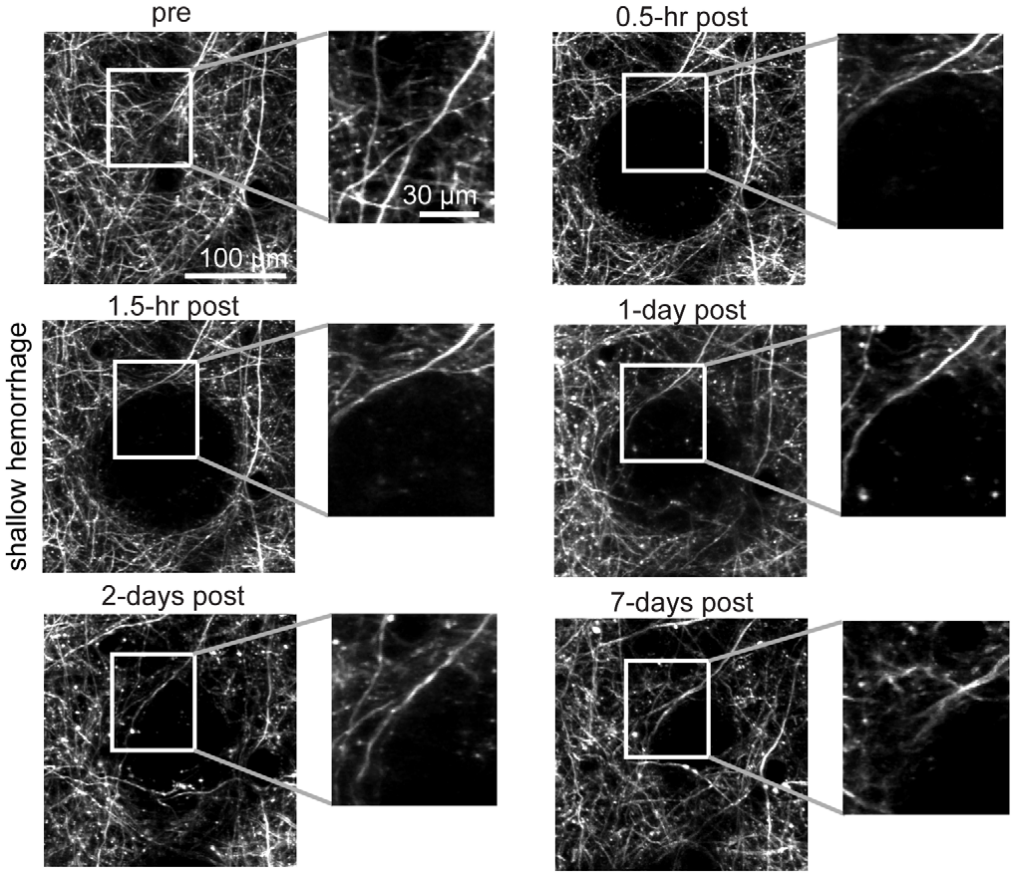
Acute and chronic imaging of dendrite morphology after shallow microhemorrhage. Maximum intensity projections of 2PEF image stacks of Layer II/III cortical dendrites from YPF-H mice at different times after microhemorrhage of a single PA 30 µm beneath the cortical surface (shallow hemorrhage).

**Figure 7:**
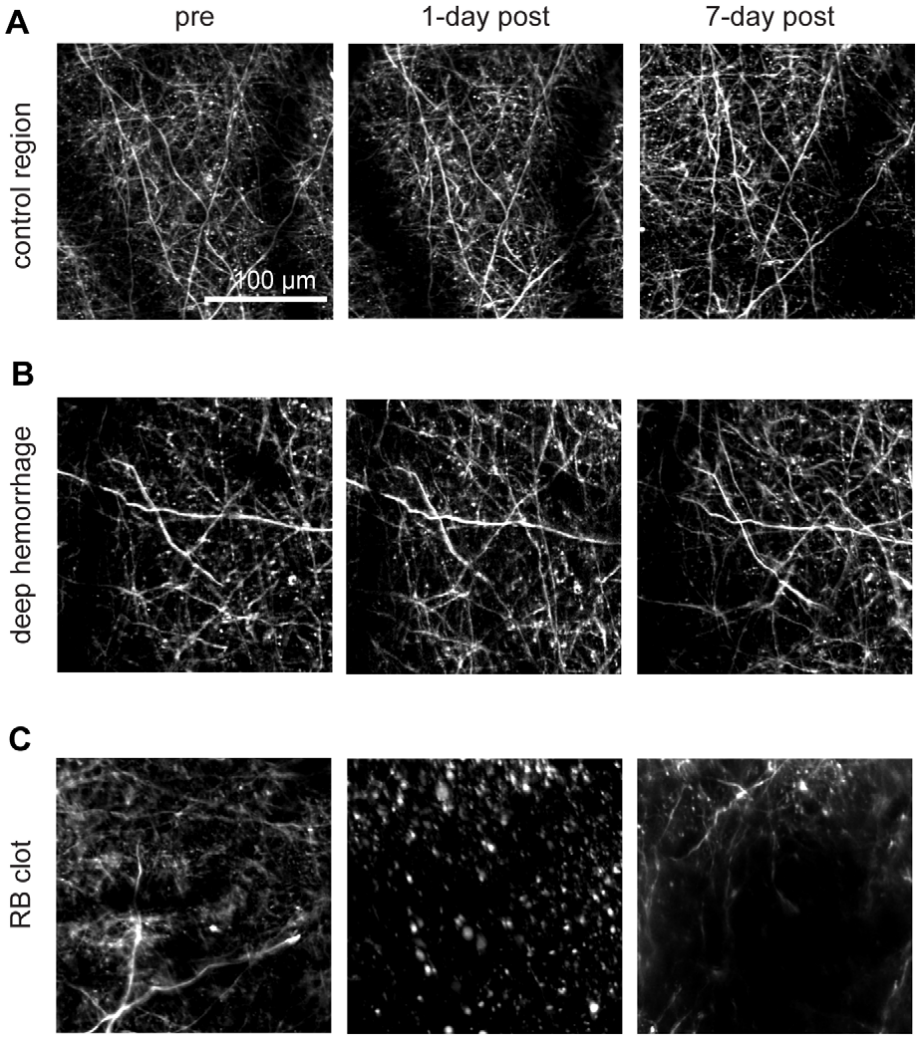
Acute and chronic imaging of dendrite morphology for controls and after a deep microhemorrhage or ischemic lesion. Maximum intensity projections of 2PEF image stacks of Layer II/III cortical dendrites in YFP-H mice (**A**) in control regions, (**B**) after microhemorrhage of a single PA at 400 µm beneath the cortical surface (deep hemorrhage), and (**C**) after photothrombotic clotting of a single PA (RB clot). For the deep hemorrhage, the RBC-filled core is not visible because it was located deep in the cortex, hundreds of micrometers directly beneath the shallow dendrites imaged here.

**Figure 8:**
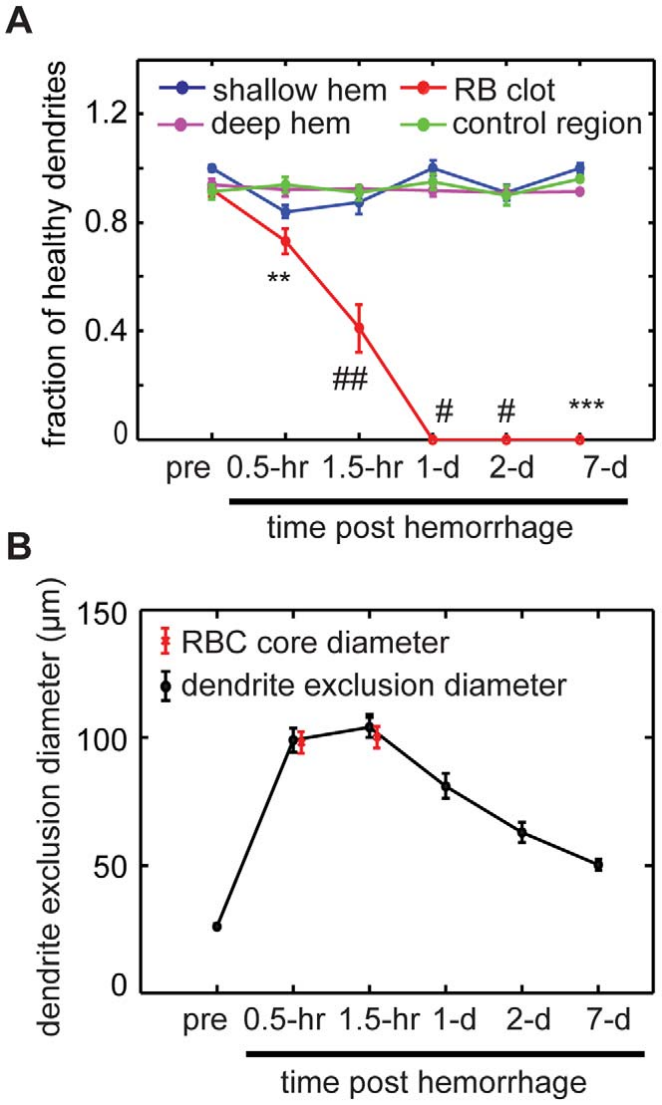
Quantification of dendrite morphology after cortical microhemorrhage. (**A**) Fraction of identified dendrites that showed no signs of degeneration as a function of time for hemorrhages produced 20–100 µm beneath the cortical surface (shallow hemorrhage), 300–500 µm beneath the surface (deep hemorrhage), for photothrombotic occlusion of a PA (RB clot), and controls. (**B**) Dendrite exclusion diameter as a function of time after microhemorrhage (black). Red symbols indicate RBC core size. Error bars represent standard error of the mean (SEM). p-value compared to controls: * p<0.05, ** p<0.01, *** p<0.001, # p<0.0001, ## p<0.00001; Mann Whitney U test.

To assess the impact of cortical microhemorrhages on neuronal health, we evaluated morphological changes characteristic of dendrite degeneration (i.e. appearance of blebs) in the apical tufts of layer V pyramidal neurons in YFP-H mice acutely and over seven days following a microhemorrhage produced within the top 100 µm of the cortex (16 hemorrhages in 10 mice; Figure 6). Immediately after the microhemorrhage, we found no degeneration in displaced dendrites surrounding the RBC-filled core (Figures 3 and 6). As noted above, for a small number of dendrites initially located immediately adjacent to the target vessel, we were unable to identify the dendrites after the microhemorrhage. It is possible that these dendrites were damaged by the laser directly or by the expanding hematoma. No degenerative changes were observed in the dendrites we could image over the following week. In addition, the dendrites shifted position back into the region occupied by RBCs immediately after the microhemorrhage, although the arrangement of the dendrites did not necessarily appear to be the same as before the lesion (Figure 6). Control regions imaged in the same animals showed no degenerative changes in dendrites (8 control regions in 7 mice; Figure 7A). We also placed microhemorrhages near the layer V neuronal cell bodies (300–500 µm below the cortical surface) and again saw no degenerative changes in dendrites (4 hemorrhages in 3 mice; Figure 7B). In contrast, photothrombotic occlusion of a single PA [16] led to a rapid and nearly complete degeneration of nearby dendrites that persisted to one week (4 clots in 3 mice; Figure 7C). The dark volume with no dendrites or fluorescent debris visible at one week suggested an ischemic infarct (Figure 7C). To quantify these observations, we determined the fraction of visible dendrites within 150 µm from the target PA that did not show any degenerative changes as a function of time. We found no differences in the number of healthy dendrites for a shallow or deep microhemorrhage as compared to controls out to seven days, while photothrombotic occlusion led to complete degeneration of all dendrites within one day (Figure 8A). In addition, we found no differences in the number of dendrites visible in each imaging volume over one week for the control, shallow, and deep microhemorrhage, indicating that in addition to visible dendrites not degenerating, there was no net loss of dendrites. In contrast, the number of visible dendrites dropped dramatically for the photothrombotic occlusion.

The extravasated RBCs from a microhemorrhage displaced dendrites, creating a dendrite-free region of 100+/−36-µm diameter (0.5-hr post-hemorrhage), consistent with the average diameter of the RBC core (Figure 8B). Beginning one day after the microhemorrhage dendrites migrated back into the region from which they were excluded, approaching the baseline exclusion diameter after seven days (Figures 6 and 8B).

**Figure 9:**
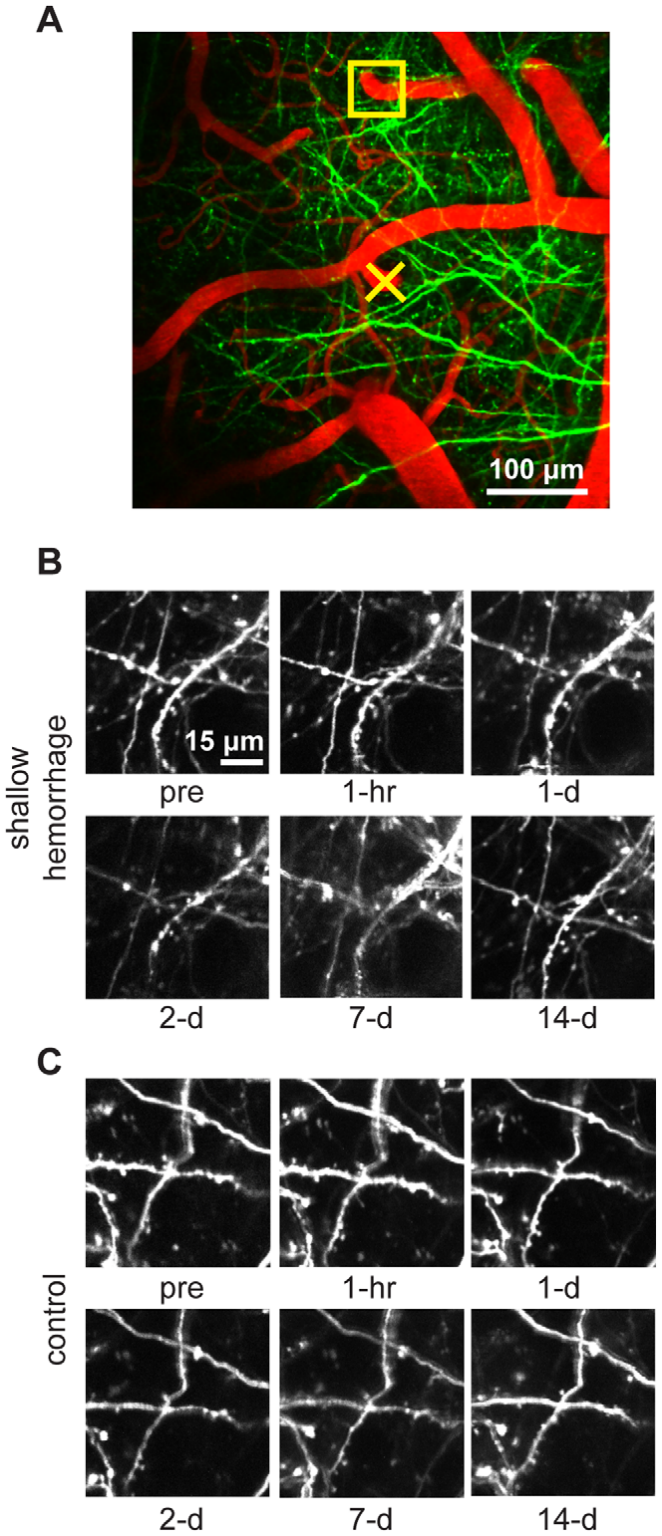
Tracking of individual dendrites over 14 days after a microhemorrhage. (**A**) 2PEF image of dendrites (green) and blood vessels (red) at basel ine in GFP-M mice. The yellow X indicates the PA that was ruptured. (**B**) 2PEF imaging of dendrites located 170 µm from the target PA (yellow box in panel A) over two weeks. (**C**) 2PEF images from a control region.

The density of labeled dendrites in the YFP-H mice prevented us from reliably tracking individual dendrites over time, making it difficult to completely rule out dendrite loss. We repeated the microhemorrhage experiments in GFP-M mice, which have much sparser dendrite labeling, enabling tracking of individual dendrites (Figure 9). Thirty-seven individual dendrites that were located within 200 µm from the target PA were identified at baseline, one hour after, then one, two, seven, and 14 days after the microhemorrhage (Figure 9B; 2 microhemorrhages across 2 mice). Control regions from the same animals also showed no loss of dendrites (Figure 9C; 68 dendrites in 2 control regions across 2 mice). In four additional animals, we imaged the dendrites less frequently and identified 99 dendrites at baseline, one hour after, and at an additional time point 5 to 14 days after the microhemorrhage. In total, across 136 individually tracked dendrites 100% were observed to neither disappear nor show degenerative changes after the microhemorrhage (95% confidence interval of 97.8% to 100%, binomial distribution). These data further indicated that microhemorrhages do not directly lead to degeneration of neurons.

### Microglia/macrophage density near a microhemorrhage increased after one day and persisted beyond a week

**Figure 10:**
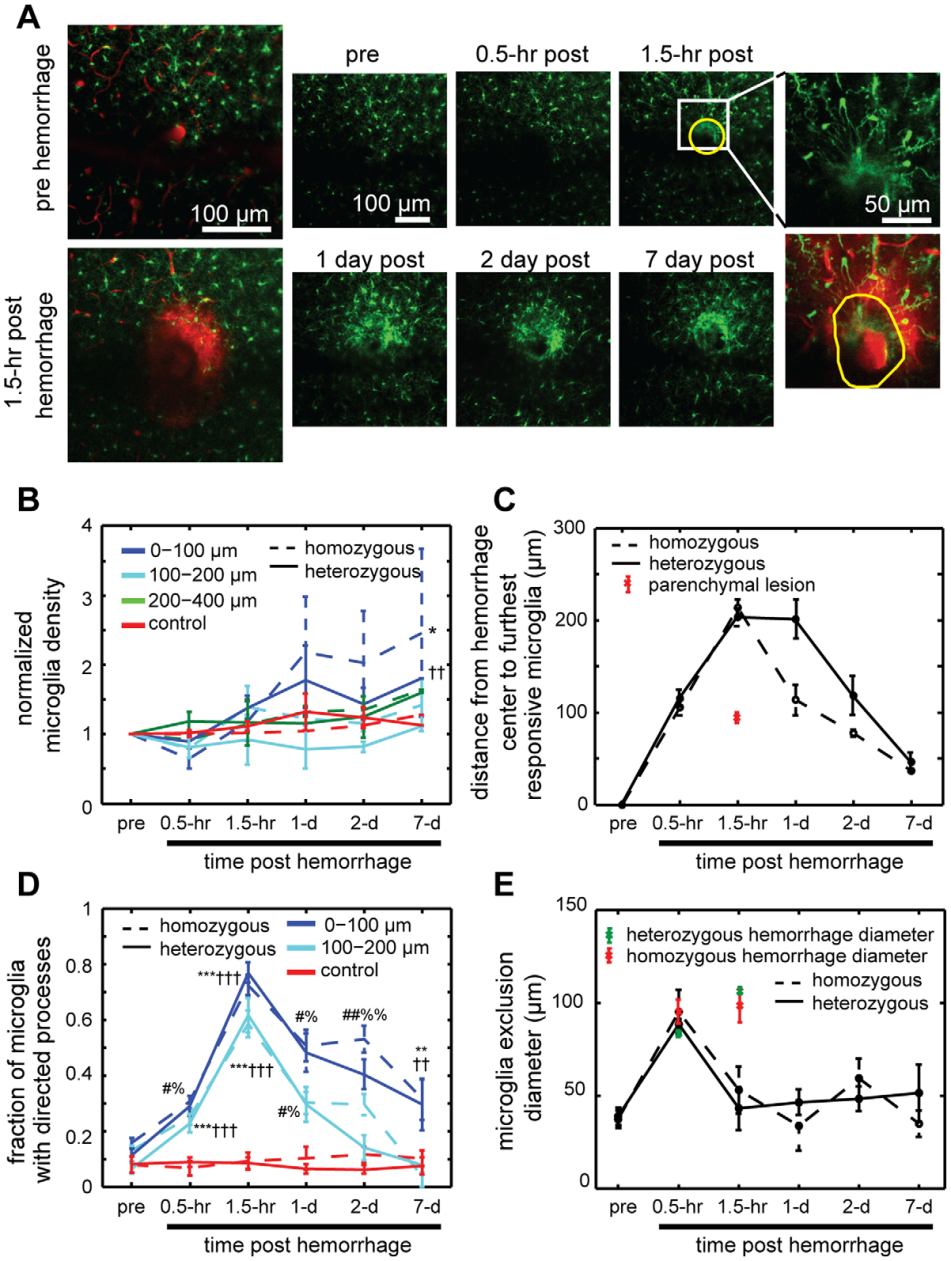
Acute and chronic imaging of microglia/macrophage response after microhemorrhage. (**A**) Maximum intensity projection of 2PEF image stacks of microglia/macrophages (green) and blood vessels (red) before and after microhemorrhage (left). Chronic dynamics of microglia/macrophage behavior after microhemorrhage (right). Inset at right shows reactive processes invading the RBC-filled core (indicated by yellow outline) at 1.5 hr after the lesion. (**B**) Microglia/macrophage density relative to baseline at different distances from the microhemorrhage as a function of time for homozygous and heterozygous CX_3_CR1-GFP mice (p-value compared to control: * <0.05, †† <0.01; analysis of covariance). (**C**) Maximal distance from the center of the microhemorrhage to the furthest responsive microglia/macrophage as a function of time after the lesion. Red symbol indicates distance to furthest responsive cell after laser ablation in the cortical parenchyma with an energy similar to that used to induce a microhemorrhage. (**D**) Fraction of microglia/macrophages with processes directed toward the lesion after a microhemorrhage (p-value compared to control for homozygous (heterozygous) mice: * (†) p<0.05, ** (††) p<0.01, *** (†††) p<0.001, # (%) p<0.0001, ## (%%) p<0.00001; Mann Whitney U test). (**E**) Diameter of region where microglia/macrophages are excluded after microhemorrhage as a function of time. Average RBC core diameter for the heterozygous (homozygous) animals is indicated in green (red) at 0.5 and 1.5 hr post hemorrhage. Error bars represent the standard error of the mean (SEM).

Next, we sought to examine the inflammatory response to cortical microhemorrhage. Both brain-resident microglia as well as blood-borne monocytes are GFP positive in the CX_3_CR1-GFP transgenic mouse [14]. The hemorrhage could potentially push monocytes directly into the brain during bleeding, and later invasion of monocytes in response to the microhemorrhage is likely. Thus, we are unable to distinguish between microglia and blood-derived macrophages in our analysis. 2PEF imaging of microglia/macrophages in heterozygous and homozygous CX_3_CR1-GFP mice (Figure 10A) revealed an increase in microglia/macrophage density within 100 µm of the lesion beginning at one day and persisting beyond one week (Figure 10B; heterozygous p<0.01, homozygous p<0.05; analysis of covariance; 5 (6) microhemorrhages in 4 (5) heterozygous (homozygous) mice). Because we found some difficulty in distinguishing individual cell bodies within about 25 µm from the edge of the RBC core where the cell density was highest, the increases in microglia/macrophage density reported here represent a conservative estimate. Microglia/macrophage density neither increased nor decreased at larger distances from the microhemorrhage.

### Microglia/macrophages rapidly responded to the microhemorrhage by directing processes toward the injury and into the RBC-filled core

We next analyzed the spatial scale and temporal dynamics of microglia/macrophage response after microhemorrhage. We measured the distance from the center of the hemorrhage to the farthest responsive cell, defined as a microglia/macrophage with more than half of its processes directed towards the site of injury. Within 1.5 hours, cells out to 200 µm from the center of the microhemorrhage were responsive for both heterozygous and homozygous mice and this distance decreased over a week (Figure 10C). In addition, we found that the fraction of microglia/macrophages near the microhemorrhage that responded peaked at 1.5 hours after the lesion (∼75% responding) and decreased over the following week (Figure 10D). To decouple the microglia/macrophage response due to laser ablation from the response due to the extravasation of blood components, we performed parenchymal tissue ablation using a laser energy similar to that used to induce a microhemorrhage. We found that the furthest responsive microglia was about 90 µm from the injury (9 ablations in 3 heterozygous mouse), suggesting that the response of microglia/macrophages after a laser-induced microhemorrhage is dependent on bleeding into the brain parenchyma, not just the laser ablation.

Microglia/macrophage cell bodies were displaced by extravasated RBCs immediately after the microhemorrhage. However, in both heterozygous and homozygous mice, microglia/macrophages extended processes into the RBC core as early as 1.5 hours after the microhemorrhage (Figure 10A and 10E), further indicating rapid cellular response to the lesion.

### The fractalkine receptor, CX_3_CR1, does not play a role in microglia/macrophage activation after microhemorrhage

We did not observe significant differences in the acute microglia/macrophage response nor in the long term increase in microglia/macrophage density between animals that had one functional copy of CX_3_CR1 (heterozygous) and animals where this receptor is knocked out (homozygous). This data suggests that other signaling pathways are responsible for microglia activation and macrophage recruitment after a cortical microhemorrhage.

### GFAP was upregulated in astrocytes seven days after microhemorrhage but no infarction was observed

**Figure 11:**
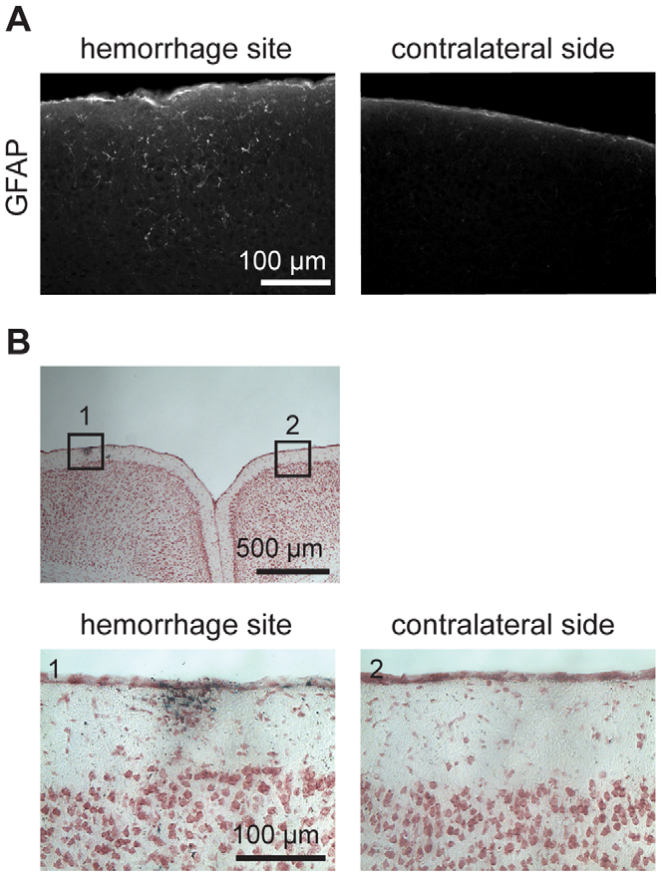
Astrocyte activation and RBC breakdown products seven days after microhemorrhage. (**A**) Immunohistology for GFAP in coronally-sectioned tissue at lesion site and contralateral control region. (**B**) Bright-field image of coronally-sectioned tissue stained with cresyl violet (pink; neuronal cell bodies) and Prussian blue (black; RBC breakdown products).

To corroborate our findings of increased inflammation with no neuron degeneration after a microhemorrhage, we analyzed astrocyte activation and tissue structure using post-mortem histology. Seven days after microhemorrhage, mice were euthanized and perfused (16 microhemorrhages in 6 mice). GFAP immunohistology showed astrocyte activation, indicative of an inflammatory response, in cells up to about 200 µm from the microhemorrhage (Figure 11A). Punctate, iron deposits spanning a diameter of ∼100 µm were observed using Prussian blue staining, but no loss of neurons (visualized by Nissl staining) or tissue infarction was seen (Figure 11B).

## Discussion

We used femtosecond laser ablation to rupture targeted PAs in mice to mimic the 100-µm sized microhemorrhages found to be correlated with dementia and cognitive decline in humans [11], [21], [22]. We then followed the dynamics of multiple cellular constituents of the brain using chronic 2PEF imaging, allowing us to determine the physiological consequences of a microhemorrhage over seconds to weeks after the lesion was created. Immediately after microhemorrhage, RBCs and blood plasma extravasated 50 µm and 250 µm, respectively, from the ruptured vessel into the parenchymal tissue (Figure 1). Microglia/macrophages responded within hours up to 200 µm from the microhemorrhage and sent cellular processes into the RBC core (Figure 10). The tissue compression from the extravasated RBCs displaced cortical dendrites (Figures 3 and 4) and microglia, but did not lead to degeneration of dendritic arbors either immediately or over the following week (Figures 6, 8, and 9). Only minor decreases in blood flow in nearby brain capillaries were observed (Figure 5). Over days following the microhemorrhage, cortical dendrites returned to the region that was filled with RBCs (Figures 6 and 8), microglia/macrophage density within 100 µm of the lesion increased, while the number of responsive microglia/macrophages outside this region decreased toward baseline (Figure 10).

### Microvascular bleeding is limited by clotting of the ruptured vessel

The spatial extent of the bleeding after a microhemorrhage could be limited by a balance between the pressure in the ruptured vessel and the contact stress of the RBC core with the parenchymal tissue. However, we found the contact stress between the RBC core and surrounding tissue to be 1.5 kPa, or about 11 mmHg, well below the typical pressure of a cortical PA. On the other hand, we found that intravascular infusion of heparin during microhemorrhage as well as additional laser irradiation of the target vessel after initial vessel rupture both led to an increase in RBC core size (Figure 2), suggesting that bleeding is limited by clotting of the vessel wall. Anticoagulant therapies, such as warfarin, are known to increase the size of ICH in patients [23], and in recent animal studies, we have shown that warfarin also increases the size of microhemorrhages [24].

### Tissue compression due to the RBC core does not result in acute ischemia

Previous studies of acute blood flow changes after ICH have found decreased CBF in the tissue around the hematoma, but it remains unclear whether this decreased CBF leads to ischemic infarction [25] or not [5], [26], [27]. For the microhemorrhages studied here, it is unlikely that there is ischemia surrounding the lesion. We found that nearly all brain capillaries within 75 µm of the hematoma core remained patent and flowing after the lesion, although the flow speed was reduced 20% in these vessels, on average. Further, the contact stress at the RBC core/tissue border was significantly less than the expected intraluminal pressure of cerebral vessels (50 (25) mmHg for arterioles (venules) [28]), suggesting that the tissue compression is not sufficient to crush nearby blood vessels.

### Microglia/macrophage response after microhemorrhage is rapid and persists locally over days

We showed an increase in the number of responsive microglia/macrophages within 200 µm of a microhemorrhage at 1.5 hours after the lesion. Other investigators have observed similar microglial responses in cells very close to an injury to the parenchymal tissue produced by irradiation with a train of high repetition rate, low-energy femtosecond laser pulses [29], [30]. A similar, rapid response in nearby microglia was also observed after the laser-induced injury to a cortical blood vessel that induced very limited bleeding [31]. The 400-µm diameter region over which microglia/macrophages respond after the microhemorrhages produced in this work correlates with the spatial extent of the extravasated blood plasma, suggesting that blood plasma components may trigger the microglia/macrophage activity.

We further observed that microglia/macrophage processes invaded the RBC core within 1.5 hours after microhemorrhage. Past work has shown that gradients in extracellular ATP [29] or chemokines released from neurons [32] cause microglia to extend processes toward focal injuries within minutes. In the case of a microhemorrhage, blood components such as fibrin, thrombin, RBC breakdown products, and blood plasma may induce cells to extend processes into the RBC core [33], [34].

We observed an increase in microglia/macrophage density within 100 µm of the microhemorrhage but no decrease in density farther from the lesion, suggesting that the increase in cell density is not due to microglia migration but rather to proliferation of brain-resident microglia or infiltration of blood-borne monocytes. Consistent with this, past work has shown proliferation of resident microglia two to three days following brain injury that leads to a three to six fold increase in the microglia population [35]. Previous work has also shown that blood-borne monocytes cross the blood-brain-barrier near the site of injury and differentiate into macrophages after brain injury [36], [37], [38].

Our experiments reveal similar microglia/macrophage response to a microhemorrhage for heterozygous and homozygous CX_3_CR1-GFP mice. Past work has shown that mice lacking functional CX_3_CR1 receptors exhibit a decrease in microglia recruitment after focal ischemia that leads to reductions in infarct volume, blood-brain barrier damage, and inflammation [39]. Similarly, less dendrite loss following mechanical injury was observed in CX_3_CR1-null animals [40]. In addition, a decrease in the number of amyloid plaques [41] as well as a decrease in neuronal loss [42] has been observed in CX_3_CR1-null mouse models of Alzheimer's disease. In contrast, our work reveals that lack of the CX_3_CR1 receptor did not dramatically change the microglia/macrophage recruitment and response after microhemorrhage and that other signaling mechanisms may be involved. We cannot, however, rule out the possibility of a different response in microglia that contain two active copies of the CX_3_CR1 receptor.

### Despite multiple potential cell injury mechanisms, we observed no structural degeneration of dendritic arbors

In vivo imaging or post-mortem assessment of dendrite morphology is a sensitive assay of cell damage that has been used to investigate the spatial scale and temporal dynamics of neuronal injury after focal ischemia [43], [44], [45], [46]. We found no evidence of degeneration in the vast majority of the imaged dendrites up to two weeks after microhemorrhage and observed a return of dendrites to the region initially filled by RBCs beginning one day after the microhemorrhage. No degeneration of layer II/III dendrites was observed for microhemorrhages placed either in the dendrite tuft or near the cell bodies (in layer V). These findings are consistent with the observation that leakage of blood plasma into the parenchyma in the periphery of an ischemic lesion was not associated with dendrite degeneration [12]. Similar to previous studies [19], our results did show dendrite blebbing following photothrombotic occlusion of a PA. A small number of dendrites that were located immediately adjacent to the target vessel were observed to acutely disappear during the expansion of the hematoma. This could be due to rapid damage to these dendrites or, perhaps, to a loss of fluorescence signal from them due to disruption of the imaging by the densely packed (and highly absorbing) RBCs in the hematoma. While we cannot determine the fate of these dendrites, for the dendrites that we were able to visualize after the hematoma had quit expanding, many of which were located very close to the hematoma core, we did not observe signs of degeneration. Taken together, this data suggest that there is very little direct damage to dendrites by a microhemorrhage, even in the region very close to the hematoma core and in the region where blood plasma has permeated the extracellular space.

Several mechanisms of brain cell injury following large ICH have been identified that could play a role after microhemorrhage. Blood components that enter the brain during bleeding, such as thrombin and plasminogen, are potentially toxic to neurons and other cells [47]. Neuronal injury can also result from excitotoxicity caused by the sudden increase in extracellular glutamate originating from the blood [6], although other work has suggested that large increases in extracellular glutamate do not lead to dendrite degeneration [12]. RBC lysis products are observed as early as one day after hemorrhage and can cause cellular injury through oxidative stress [4], [48], [49], [50]. The lack of dendrite pathology we observed suggests that individual microhemorrhages are not sufficient to cause significant cellular damage through these processes. Our experiments, however, did not examine changes in neuronal function, which may be altered even though structure is maintained. While our data shows that the large-scale structure of dendritic arbors does not degenerate, it is possible that more subtle alterations in dendrites, such as elevated rates of dendritic spine turnover or loss, are occurring in the vicinity of the microhemorrhage. If present, such spine loss or turnover would effectively rewire neural circuits and could contribute to the cognitive effects of a microhemorrhage. In addition, similar sized microhemorrhages could lead to direct neuronal damage in other brain regions, such as in white matter, which is more poorly perfused than the cortical grey matter studied here. Finally, our experiments determined the impact of microhemorrhages in healthy adult mice. It is possible that when combined with other disease states, such as amyloid plaque buildup and cerebral amyloid angiopathy or hypertension, or when occurring in an older brain microhemorrhages could cause more severe effects on nearby neurons. Our hemorrhage induction technique does not lead to the formation of a clot that blocks blood flow in the targeted vessel, so there is no ischemia produced. In naturally occurring microhemorrhages, it is possible that the clotting that stops the bleeding may sometimes also occlude the vessel lumen. In this case, we would expect more severe tissue damage from the resulting decrease in local blood flow.

### Conclusions

Taken together, our data show that microhemorrhages create a perihematomal region distinguished by a local but sustained inflammatory response, and by the absence of ischemia or widespread dendrite degeneration. The inflammation is characterized by acute activation and chronic increase in microglia/macrophages, as well as activation of astrocytes. The spatial extent of this perihematomal region is coincident with the initial distance blood plasma pushes into the brain parenchyma, suggesting that acute exposure to blood plasma components may initiate the inflammatory process. This inflammation, in turn, may be a long-term driver of neural dysfunction or death that underlies the cognitive decline linked to the accumulation of microhemorrhages. For example, inflammation has been shown to affect dendrite spine turnover in animal models of ischemia [51] and after mild brain injury [15], [52]. However, our work also shows that occlusion of individual microvessels leads to more severe neuropathology than bleeding from these vessels. Thus the cost of small vessel bleeds appears to be smaller than the cost of such vessels becoming occluded, suggesting that patients with elevated cardiovascular risk factors, such as hypertension, would potentially benefit from therapeutic strategies that prevent small vessel occlusions, even if at the expense of small vessel bleeds.
